# The Kocher-Langenbeck approach combined with robot-aided percutaneous anterior column screw fixation for transverse-oriented acetabular fractures: a retrospective study

**DOI:** 10.1186/s12891-022-05313-w

**Published:** 2022-04-11

**Authors:** Zhao-jie Liu, Ya Gu, Jian Jia

**Affiliations:** Department of Orthopaedics, Tianjin Hospital, 406 Jiefangnan Road, Tianjin, 300211 China

**Keywords:** Robotics, Percutaneous screw, Minimally invasive surgical procedures, Acetabulum, Fracture Fixation, internal

## Abstract

**Objective:**

Transverse-oriented acetabular fractures (TOAFs), including transverse, transverse with posterior wall and T-shaped fractures, are always challenging for double-column reduction and fixation with minimally invasive method. The purpose of this study is to compare the therapeutic effects of robot-aided percutaneous anterior column screw fixation versus minimally invasive anterior plate fixation for TOAFs based on the Kocher-Langenbeck (K-L) approach.

**Methods:**

Patients suffering TOAFs that were fixed by robot-aided percutaneous anterior column screw fixation or minimally invasive anterior plate fixation associated with posterior fixation via the K-L approach were divided into two groups: group A (screw fixation) and group B (plate fixation). Surgical time, blood loss, incision length for anterior fixation and complications were recorded. Fracture reduction quality was evaluated using criteria described by Matta. Fracture healing was assessed on the series of pelvic radiographs at each follow-up. Functional outcomes were investigated using the modified Postel Merle D’Aubigne score at the final follow-up.

**Results:**

Twenty-nine patients with TOAFs, including 12 patients in group A and 17 patients in group B, were evaluated for study eligibility. The mean surgical time of anterior fracture fixation was 18.7 ± 4.6 min in group A and 33.4 ± 5.0 min in group B (*P* < 0.001). The amount of intraoperative blood loss was 615.6 ± 178.7 ml in group A and 719.3 ± 199.0 ml in group B (*P* < 0.001). Incision length for anterior fixation was 9.0 ± 1.8 mm in group A and 81.2 ± 7.3 mm in group B (*P* < 0.001). The complications related to the surgery of anterior column only occurred in group B (lateral femoral cutaneous nerve palsy in 1 patient and groin discomfort in 1 patient). No significant differences in reduction quality, hospital stay, fracture healing time and functional results were noted between the two groups.

**Conclusion:**

The K-L approach combined with robot-aided anterior column screw fixation is a safe and effective option for TOAFs. Compared with minimally invasive anterior plate fixation, robot-aided screw fixation has obvious advantages on surgical time, blood loss, and invasiveness. The K-L approach combined with minimally invasive anterior plate fixation can also be a reliable alternative for TOAFs, with the similar reduction quality and functional results.

## Introduction

Transverse-oriented acetabular fractures (TOAFs), including transverse, transverse with posterior wall and T-shaped fractures, are relatively common patterns, accounting for approximately 32–46% of all the acetabular fractures [1–4]. The main transverse fracture line running in the sagittal direction can split the acetabulum from the dome to the quadrilateral surface. Due to the variable fracture lines, transverse acetabular fracture can be distinguished into the transtectal, juxtatectal or infratectal types [1].

The posterior or central displacement of a femoral head determines whether the posterior wall is involved in the transverse acetabular fracture. Because of the convenience of intraoperative exposure or the existence of posterior wall fractures, the majority of surgeons prefer the Kocher-Langenbeck (K-L) approach to treat TOAFs [5–7]. Letournel and Judet [1] reported the reduction quality of 117 patients with transverse and posterior wall fractures, of which 90 patients were treated operatively via the K-L approach with the overall perfect reduction rate of 67.5%. In a series of 60 patients with transverse and posterior wall fractures reported by Matta [8], 46 patients were treated using the K-L approach with the anatomic reduction rate of 80%.

Compared to the quality of the transverse fracture reduction through a single K-L approach in the prone or lateral position, some studies showed that the displacement of anterior column persistently existed with the mean gap of 1.3–2.1 mm and the maximal displacement of 7 mm no matter which position was chosen [9, 10]. A recent study demonstrated that the development of osteoarthritis increases significantly if the residual gap or step of the transverse acetabular fractures are more than 3 mm and 1 mm, respectively [11]. In consequence, it’s of great importance to reduce both-column simultaneously during the intraoperative procedure.

Similar to all intra-articular fractures, the key points to achieve satisfactory prognosis of acetabular fracture are to reconstruct the precise congruence of acetabulum and femoral head, restore the integrity of articular surface, and provide strong and effective internal fixation strength for early rehabilitation. The simple posterior plating probably leads to opening of the anterior fracture gap due to the unreasonable pre-contouring of the plate or failure of anterior reduction. Consequently, the K-L approach combined with an anterior fixation for transverse acetabular fractures is recommended to achieve anatomical reduction and stable fixation [12]. The stiffest fixation method for the TOAFs shown in the clinical and biomechanical studies is the combination of posterior column plates with an anterior column screw, with loss of reduction ranging between 3 and 5% [13–15]. A recent study reported a biomechanical comparison on transverse acetabular fracture stabilization with five different fixation methods (anterior plating only, posterior plating only, anterior plating plus a posterior column screw, posterior plating plus an anterior column screw and anterior plus posterior plating), and the conclusion was that internal fixation of a single column might not provide adequate stability, while strength of the plate plus column screw fixation and double plate fixation was comparable [16]. Therefore, the combination of posterior plating and percutaneous anterior column screw fixation is more reasonable, while avoiding the additional invasiveness caused by anterior approach. However, anterograde anterior column screw fixation with free-hand technique is not easy to be inserted accurately even for the experienced surgeons. Due to the uncertain entry point and the narrow bony corridor, it usually takes much time to insert the guiding wire accurately, with the consequence of excessive radiation exposure to patients and surgeons. Percutaneous fixation under the guidance of orthopedic robots has been reported with the advantages of accuracy and convenience [17, 18]. Most important of all, robot-aided technology can also assist surgeons to plan, simulate and correct the trajectory of screws intraoperatively [17, 18]. So far, the percutaneous anterior column screw fixation is possibly to be an optimal choice for the patients suffering TOAFs, especially with transverse and transverse with posterior wall fractures.

The third generation of Chinese manufactured orthopedic robot, TiRobot system, has been applied in our institution. Consequently, patients suffering TOAFs were surgically treated using the K-L approach combined with percutaneous anterograde anterior column screw fixation under robotic guidance, compared with minimally invasive anterior plate fixation based on the K-L approach. The purposes of this study are to analyze the advantages of the K-L approach combined with anterior fixation for TOAFs, summarize the key points of two methods, and evaluate the clinical and radiological results of the two methods for TOAFs.

## Patients and methods

### Inclusion and exclusion criteria

Inclusion criteria were as follows: (1) TOAFs including transverse, transverse with posterior wall and T-shaped fractures, (2) patients with TOAFs treated with posterior fixation via the K-L approach combined with anterograde anterior column screw fixation under robotic guidance, and (3) patients with TOAFs treated with posterior fixation via the K-L approach combined with minimally invasive anterior plate fixation. Exclusion criteria were as follows: (1) the concomitant ipsilateral femoral head fractures, (2) ipsilateral hip dysfunction or deformity existed in the past, (3) premature or pathological fractures, (4) acetabular fractures with obvious callus formation, and (5) the follow-up time was less than 12 months.

We retrospectively analyzed all patients with TOAFs treated in our department from October 2013 to March 2020. According to inclusion and exclusion criteria, 29 patients with TOAFs were identified and divided into two groups: the group of posterior fixation via the K-L approach combined with anterograde anterior column screw fixation under robotic guidance (group A) and the group of posterior fixation via the K-L approach combined with minimally invasive anterior plate fixation (group B). 

### Surgical Equipment and Instrument

**Fig. 1:**
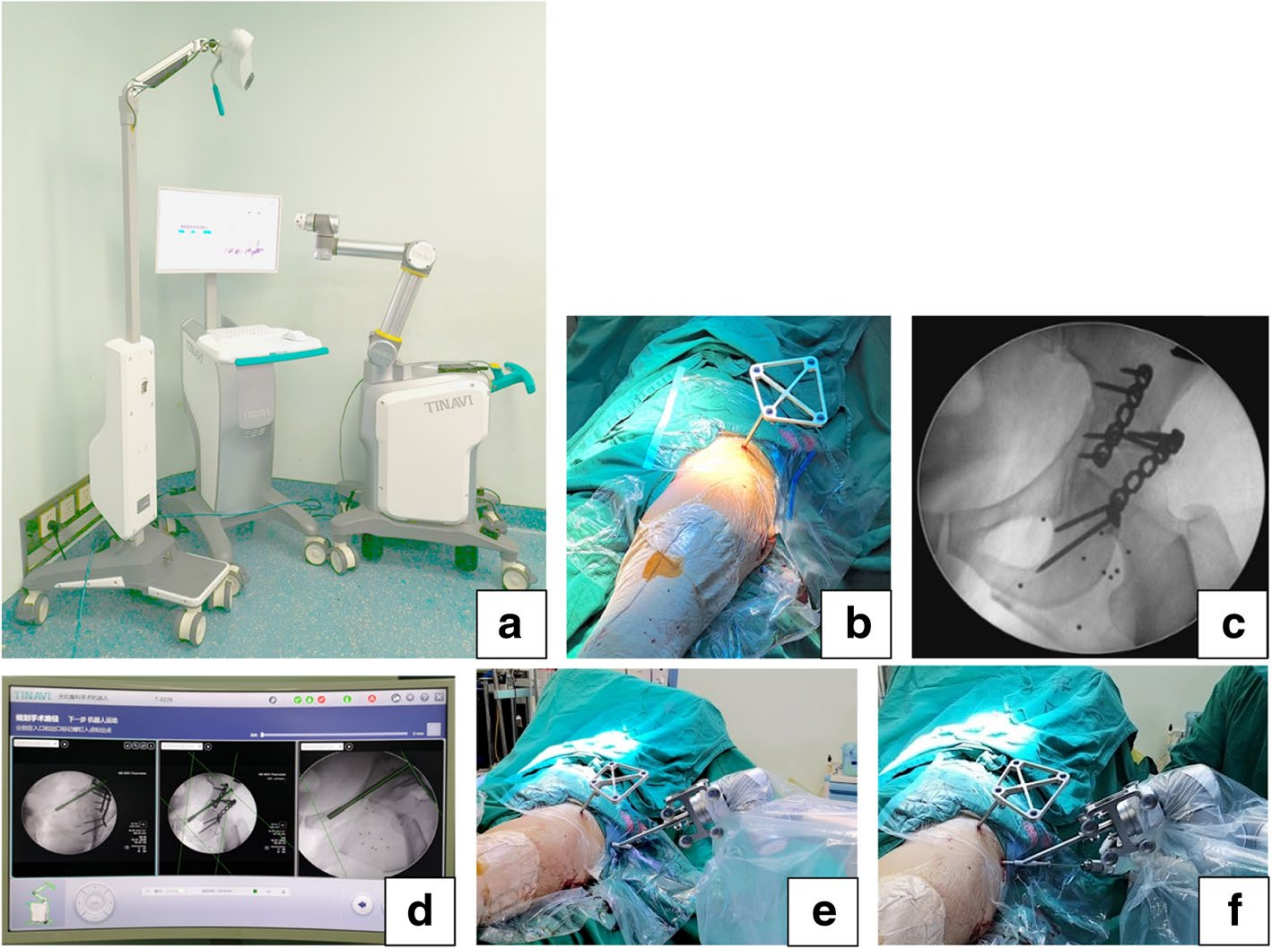
Auxiliary operation process of the TiRobot orthopedic robot. **a** The TiRobot orthopedic robot system; **b** The navigation tracker is fixed on the ipsilateral ASIS; **c** ten positioning points on the locator are shown in the view; **d** The simulation of screw placement; **e** The sleeve carried by the robotic arm is located at the target area; F The guiding wire is being drilled into the anterior column corridor along the sleeve

The TiRobot system, the third generation TianJi robot for orthopaedic surgery (TINAVI Medical Technologies, Beijing, China), is composed of a main console with surgical planning and controlling software, an optical tracking system, and a robotic arm with six-axis-arm carrying a navigation and positioning toolkit (Fig. 1a). Additional surgical equipment includes a C-arm machine (Siemens, Germany).

### Surgical technique

#### Preoperative management

According to the advanced trauma life support (ATLS) guidelines, initial stabilization of vital signs was urgently performed after hospital admission. Resuscitation with fluid and blood transfusion in the emergency department was undertaken if necessary. Posterior hip dislocations were promptly reduced with closed technique after hemodynamic stabilities. Ipsilateral skeletal tractions were performed in all patients to maintain the stabilization of the affected hip joint. Preoperative radiographs and computed tomography (CT) scans were routinely acquired. Once the patient’s physiological condition was stable, definitive surgeries were performed.

#### Surgical procedures

Intravenous antibiotics were administered within 30 min of the skin incisions. Following administration of general anesthesia, cleaning and draping of the affected lower extremity and pelvic area were completed in the floating position on a radiolucent table. All surgical procedures were performed by the same surgical team with sufficient experience.

The first part of the acetabular fracture management was similar in almost all the patients with a standard K-L approach in the lateral position. The posterior column and posterior wall fractures were exposed under direct vision, together with palpation of the anterior fractures through the greater sciatic notch. The hematoma, embedded soft tissue and bone callus at the fracture site were removed to expose the primary fracture ends. Manual traction of the affected lower limb makes it easier for the surgeon to remove the isolated fragments in the articular cavity. A φ6-mm threaded pin was drilled into the ischial tuberosity and a φ4.5-mm screw was respectively inserted into both sides of the fracture end. Then we connected the threaded pin with a “T” handle as a “joy stick” to correct the rotational displacement of the quadrilateral surface and used a Jungbluth clamp to eliminate fracture gaps by gripping the reduction screw heads simultaneously, ensuring the anatomical reduction of both columns. If necessary, angled forceps can be applied to clamp the quadrilateral surface or even the anterior column through the greater sciatic notch. After posterior column component had been fixed with Kirshner wires temporarily, the femoral head was utilized as a template for the further reduction of the posterior wall fracture. Cancellous autograft bone harvested from the greater trochanter or allograft bone needs to be used if the acetabular fracture is associated with compression of articular surface. Once anatomical reduction and congruence were achieved, the posterior column and posterior wall fractures were fixed with one or two 3.5 mm pre-contoured reconstruction plates. Next, the anterior column involved by transverse fracture line was managed with different methods as follows.

**Fig. 2:**
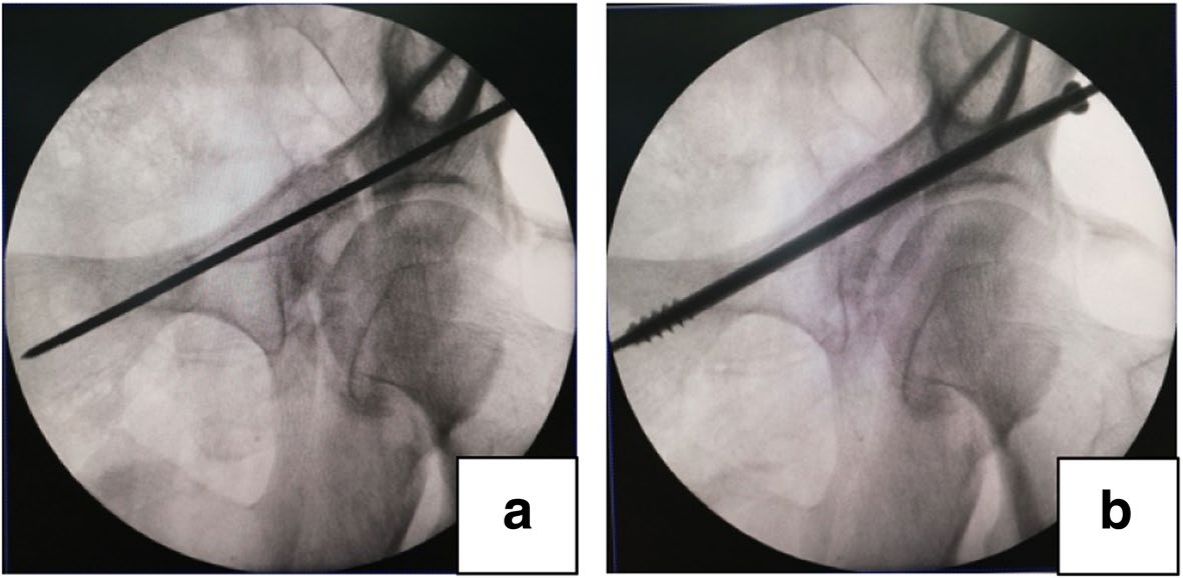
The compression effect of anterior column screw fixation; **a** An obvious fracture gap at the anterior column; **b** The gap disappearing after anterior column screw fixation

For patients in group A, anterograde anterior column screw fixation was performed under robotic guidance. Due to difficulties of achieving standard fluoroscopic views in the lateral position, the supine position can be selected to facilitate the manipulation of screw insertion. A navigation tracker was fixed into ipsilateral anterior superior iliac spine (ASIS) percutaneously (Fig. 1b). After a sterile working environment for the robotic arm had been established by covering disposable plastic coat, the locator was assembled and connected with the end of the robotic arm. Next, fluoroscopic pelvic images taken by C-arm machine (anteroposterior, iliac oblique, and obturator oblique views) were obtained. After all the ten positioning points on the locator were shown in the views, the images were transmitted to the main console (Fig. 1c). Then, the length, angulation and direction of an anterograde anterior column screw were planned and the simulation of screw placement was evaluated (Fig. 1d). Once the command is given, the robotic arm would move to the target area according to the planned trajectory outside the body (Fig. 1e). When the sleeve arrived at the planned entry point through a 1 cm incision, the screw trajectory was recalibrated. At that moment, what the surgeon needed to do was to drill the guiding wire along the sleeve instead of looking for the accurate entry point repeatedly under the C-arm machine (Fig. 1f). In this process, the robot can monitor the needle in real time. Finally, anterior column lag screw fixation was performed using a φ6.5-mm partially threaded cannulated screw to eliminate the anterior fracture gap. After fixation, we flexed and rotated the hip joint to check the fixation stability of the fracture and determine if there was friction or snapping in the joint. In the end, anteroposterior, inlet and obturator outlet radiographic views were taken to confirm that the cannulated screw was completely in the bony corridor and didn’t penetrate into the hip joint. If there were only gap without step and rotation of the anterior column fracture, percutaneous anterior column screw fixation can be inserted prior to posterior fixation (Fig. 2a, b).

**Fig. 3:**
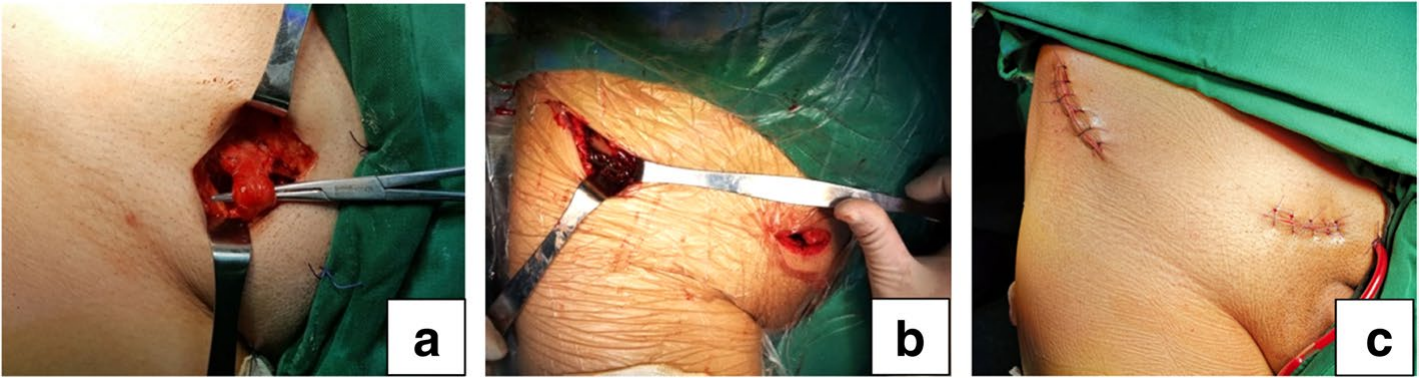
Minimally invasive anterior plate fixation; **a** Protection of the spermatic cord in the medial incision; **b** Plate insertion and fixation; **c** Anterior sutured incisions

For patients in group B, the following procedures must be performed in the supine position. Two small anterior incisions, a medial one and a lateral one, were made for the plating insertion and fixation. The medial incision, length of 3 to 4 cm, was made along the ipsilateral superior pubic ramus. The spermatic cords in male patients or round ligaments of the uterus in female patients were protected (Fig. 3a). Deep fascia was separated underneath the inguinal ligament, and the external iliac blood vessels were palpated and distracted laterally to prevent iatrogenic injuries. The pectineal muscle was detached to expose the superior ramus of the pubis. The lateral incision, length of 4 to 5 cm, was centered on the anterior superior iliac spine and curved along the iliac crest. Lateral femoral cutaneous nerve must be identified and protected. Then origins of abdominal muscle at iliac internal crest were sharply detached. With hip flexion at about 60°, further blunt subperiosteal elevation of the iliopsoas and neurovascular bundle was performed with a periosteal detacher through the two incisions, creating a subperiosteal tunnel. A 3.5 mm 12 holes pre-contoured reconstruction plate was inserted along the prepared subperiosteal tunnel from the lateral incision and placed on the superior part of the ramus to enable screw placement towards the ischiopubic rami. Laterally, the plate was close to the anterior superior and inferior iliac spine (Fig. 3b). Several reduction tools such as ball spike pusher were necessarily used in increasing direct contact between the plate and the bone. Initially three screws through medial holes were fixed, and then the plate was distracted laterally as much as possible by holding the end of the plate and anterior superior iliac spine with forceps. A lag screw was applied through a lateral hole of the plate to eliminate the anterior fracture gap, followed with the other two screws fixed through the other two lateral holes of the plate, which can provide enough stabilization for anterior part of the transverse fracture. After reduction was verified using the C-arm fluoroscopy, the wound was subsequently rinsed and sutured (Fig. 3c).

### Postoperative management and follow-up

Postoperatively, antibiotic therapy was administered routinely for 24 h, and negative pressure drainage was used for 24 to 48 h. Mechanical and medication prophylaxis against venous thrombus embolism (VTE) was applied from the first day after surgery until patient discharge. Patients began a gradual improvement of hip passive range-of-motion and quadriceps and gluteus muscles strengthening exercises once the drain was removed. Gradually, active range-of-motion exercises of the affected hip were encouraged and partial weight-bearing with crutches was maintained at least 6–8 weeks. Full weight-bearing was permitted only 3 months postoperatively when radiographs demonstrated union as evident by callus formation.

Radiographic evaluation of fracture reduction was performed for each patient instantly after surgery with the Matta’s radiological criteria by measuring the residual displacements on the postoperative radiographs [8]. The highest of the three values was used for grading according to one of the three categories: anatomic reduction (0–1 mm of displacement), imperfect reduction (2–3 mm), and poor reduction (> 3 mm). Patients were followed up at the outpatient department with standard radiographs every month until the acetabular fractures heal. At the final follow-up, clinical results of the affected hips were investigated with the modified Postel Merle D’Aubigné score system [8, 19], which was mainly evaluated from three aspects, including pain, walking, and range of activity. The scores were categorized as excellent (18 points), good (15–17 points), fair (13–14 points), or poor (<13 points). According to Brooker classification system [20], the formation of heterotopic ossification was recorded and subdivided into four categories (grades I, II, III, and IV).

### Statistical analysis

Data analysis was performed by using SPSS20.0 statistical software (SPSS Inc., Chicago, IL, USA). Quantitative data were expressed as mean ± standard deviation (SD) and were compared using the Student *t*-test. Categoric variables were compared using the Pearson *X*^2^ test. The *P*-value was set <0.05, which was considered statistically significant.

## Results

In total, 29 patients with TOAFs, including 12 patients in group A and 17 patients in group B, were assessed for study eligibility. There were similarities in age, gender, classification, posterior hip dislocation, sciatic nerve injury, preoperative time and follow-up time, with no statistical differences between the two groups on patient characteristics (Table 1).

**Table 1:** Patient characteristics of the two groups

Patient characteristics	Group A (***n*** = 12)	Group B (***n*** = 17)	Test value	***P*** value
Age (year), M ± SD	37.9 ± 12.3	38.8 ± 8.6	*t* = −0.219	0.828
Gender, n(%)			*X*^*2*^ = 0.012	0.913
Male	8 (66.7%)	11 (64.7%)		
Female	4 (33.3%)	6 (35.3%)		
Mechanism of injury			*X*^*2*^ = 1.661	0.436
Traffic injury	8 (66.7%)	11 (64.7%)		
Fall injury	1 (8.3%)	2 (11.8%)		
Crush injury	3 (25.0%)	4 (23.5%)		
Letoural &Judet classification, n(%)			*X*^*2*^ = 0.024	0.988
Transverse	4 (33.3%)	6 (66.7%)		
Transverse and posterior wall	6 (50.0%)	8 (47.1%)		
T-shaped	2 (16.7%)	3 (17.6%)		
Posterior hip dislocation, n(%)			*X*^*2*^ = 0.024	0.876
Yes	6 (50.0%)	8 (47.1%)		
No	6 (50.0%)	9 (52.9%)		
Sciatic nerve injury, n(%)			*X*^*2*^ = 0.062	0.804
Yes	1 (8.3%)	2 (11.8%)		
No	11 (91.7%)	16 (88.2%)		
Preoperative time (day), M ± SD	6.6 ± 5.1	6.2 ± 4.0	*t* = 0.240	0.812
Follow-up time	14.7 ± 3.8	16.5 ± 3.7	*t* = −1.267	0.216

*t*, Independent t test; *X*^*2*^, Chi-square test; M ± SD, mean ± standard deviation; n, patient number

The mean surgical time of anterior fracture fixation was 18.7 ± 4.6 min in group A and 33.4 ± 5.0 min in group B. The amount of intraoperative blood loss was 615.6 ± 178.7 ml in group A and 719.3 ± 199.0 ml in group B. The significant differences were revealed on above values (*P* < 0.001) (Table 2). According to the comparison of incision length for anterior fracture fixation, the average incision length in group A was 9.0 ± 1.8 mm which was only for anterior column screw insertion, while that in group B was 81.2 ± 7.3 mm which was the sum of internal and lateral incision length for anterior plate fixation. There was significant difference between the two groups (*P* < 0.001). Due to the guidance of robotic navigation, anterior column screws that were inserted percutaneously in group A were exposed to radiation with an average of 16 ± 3.5 times (range, 12–23 times) intraoperatively. However, fluoroscopy was not performed frequently in group B due to the application of anterior column plates. The mean hospital stay in group A with 14.0 ± 3.6 days compared to that in group B with 14.2 ± 2.9 days with no statistical difference (*P* = 0.419). All patients demonstrated radiological and clinical bony union within 2–6 months after surgery. The acetabular fractures healed with an average time of 3.4 ± 0.9 months in group A and 3.5 ± 1.0 months in group B, respectively (*P* = 0.769).

**Table 2:** Perioperative clinical indicators of the two groups

Patient characteristics	Group A	Group B	Test value	***P*** value
Surgical time of the anterior column fracture (min), M ± SD	18.7 ± 4.6	33.4 ± 5.0	*t* = −8.117	<0.001
Blood loss (ml), M ± SD	615.6 ± 178.7	719.3 ± 199.0	*t* = −35.309	<0.001
Incision length for anterior fixation (mm), M ± SD	9.0 ± 1.8	81.2 ± 7.3	*t* = −100.339	<0.001
Reduction quality, n(%)			*X*^*2*^ = 0.829	0.661
Anatomic reduction	10 (83.3%)	14 (82.4%)		
Imperfect reduction	2 (16.7%)	2 (11.8%)		
Poor reduction	0	1 (5.9%)		
Complications, n(%)			*X*^*2*^ = 0.514	0.773
Lateral femoral cutaneous nerve palsy	0	1		
Heterotopic ossification	1	2		
Groin discomfort	0	1		
Total rate	1 (8.3%)	4 (23.5%)		
Hospital stay (day), M ± SD	14.0 ± 3.6	14.2 ± 2.9	*t* = −0.814	0.416
Healing time (month), M ± SD	3.4 ± 0.9	3.5 ± 1.0	*t* = −0.294	0.769
Modified Postel Merle D’Aubigne score			*X*^*2*^ = 0.115	0.944
Excellent	8 (66.7%)	11 (64.7%)		
Good	3 (25.0%)	5 (29.4%)		
Fair	1 (8.3%)	1 (5.9%)		

*t*, Independent t-test; *X*^*2*^, Chi-square test; M ± SD, mean ± standard deviation; n, patient number

### The imaging and clinical results

The series of standard radiographs and CT scans of the pelvis were acquired in one week after surgery. The results revealed that all the affected hip joints treated with surgery achieved congruence, and the iliopectineal and ilioischial lines recovered the continuity. According to the criteria of reduction quality described by Matta, group A had 10 patients with anatomic reduction and 2 with imperfect reduction, compared to 14 patients with anatomic reduction, 2 patients with imperfect reduction and 1 patient with poor reduction in group B (*P* = 0.661). The maximal residual displacement was a gap with 4 mm at the anterior column, which was shown on the postoperative CT scans, belonging to poor reduction. None of the screws penetrated into the hip joint. All of the cannulated screws were located in the bony corridor of anterior column, with the mean screw length of 111.3 ± 15.3 cm (range, 85-135 mm).

According to the modified Postel Merle D’Aubigne score system [8, 19], functional outcomes achieved in group A (excellent in 8 (66.7%) patients, good in 3 (25%) patients, and fair in 1 (8.3%) patient) were similar to that in group B (excellent in 11 (64.7%) patients, good in 5 (29.4%) patients, fair in 1 (5.9%) patient) at the final follow-up, with no statistical differences (*P* = 0.944).

### Complications

**Fig. 4:**
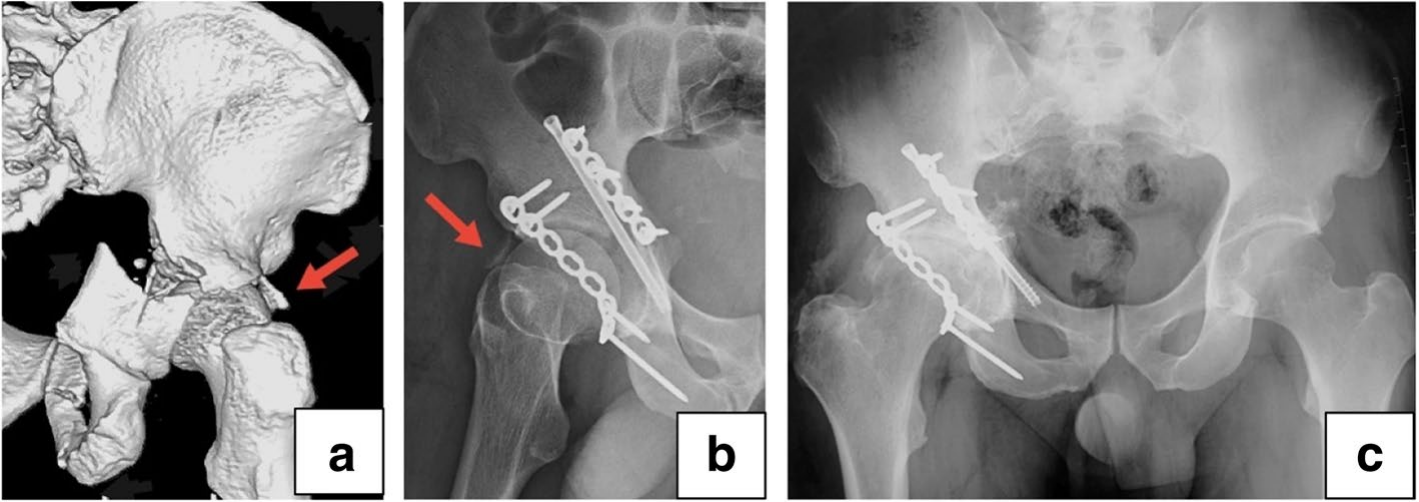
The complication of heterotopic ossification in one case. **a** An isolated fragment is located at the lateral side of the femoral head (red arrow). **b** A postoperative radiograph shows that the fragment was not removed (red arrow). **c** An antero-posterior view shows heterotopic ossification with grade II at the affected hip joint at one-year follow-up

There was no iatrogenic neurovascular damage intraoperatively. No infection and incision-related complications occurred in all patients. At the final follow-up, none of the patients had hardware failure, post-traumatic osteoarthritis or avascular necrosis of the affected femoral head. Three patients with traumatic sciatic nerve injuries (1 patient in group A and 2 patients in group B) were found to have continuous nerves during exploration, and recovered completely after taking oral neurotrophic medicine 3–6 months. Numbness in the anterolateral thigh skin was identified in 1 patient (group B) due to the intraoperative distraction of lateral femoral cutaneous nerve, and recovered in 6 months after surgery without any medication. One patient in group B complained of groin discomfort in the area of anterior plating, and the symptom faded away after fracture healing and removal of the implant. Heterotopic ossification occurred in 3 patients at the final follow-up (1 patient of grade II in group A and 2 patients of grade I in group B) (Fig. 4a-c).

**Fig. 5:**
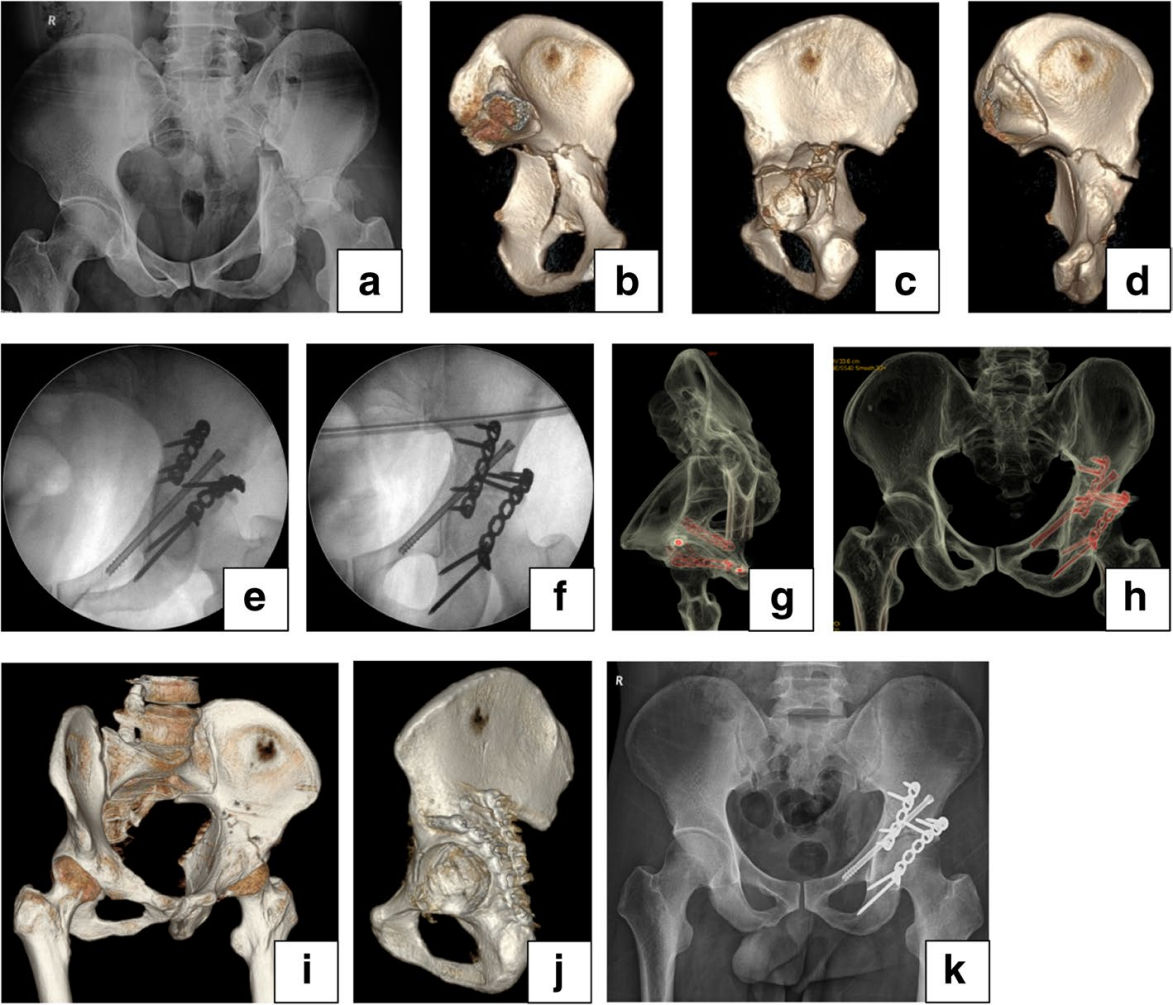
Male, 39-year-old, car accident, a T-shaped acetabular fracture associated with posterior wall fracture, treated with the K-L approach combined with percutaneous anterior column screw fixation under robotic guidance. **a** Preoperative X-ray antero-posterior image shows an acetabular fracture associated with femoral head dislocation. **b**-**d** 3D CT images of a acetabula show a T-shaped acetabular fracture associated with the communicated posterior wall. **e**, **f** Intraoperative images reveal anatomic reduction of the acetabular fracture. **g** A postoperative axial image of the anterior column demonstrates the screw is located at the bony corridor. **h**-**g**, 3D CT images show anatomic reduction of the acetabular fracture. **k** The antero-posterior X-ray image three months after surgery shows fracture healing

**Fig. 6:**
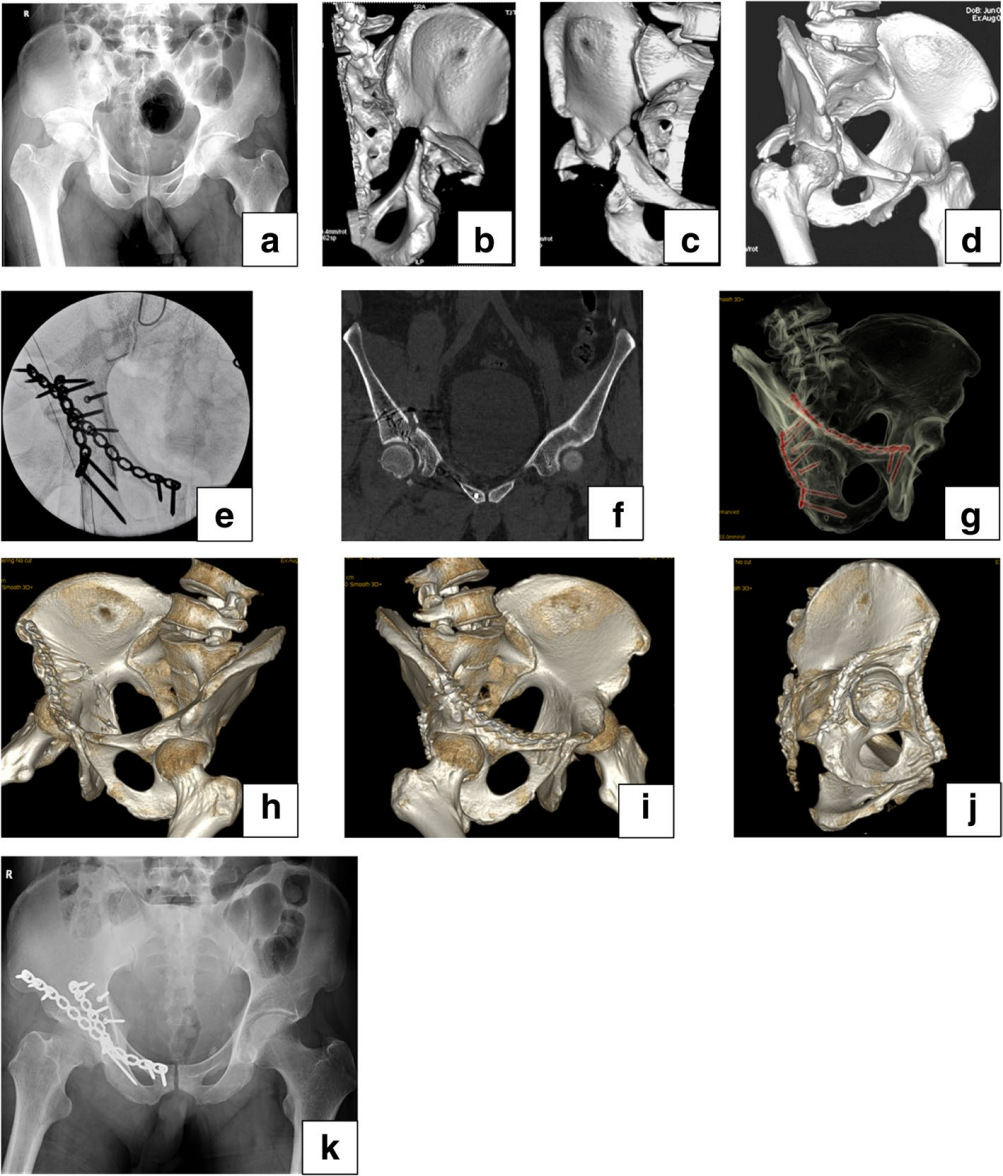
Male, 44-year-old, car accident, a transverse and posterior wall acetabular fracture, treated with posterior plate fixaiton via the K-L approach combined with minimally invasive anterior plate fixation. **a** Antero-posterior pelvic X-ray shows an acetabular fracture associated with femoral head dislocation. **b**-**d** 3D CT images show a transverse and posterior wall acetabular fracture. **e** Intraoperative views reveal the screws didn’t penetrate into the hip joint. **f**-**j** Postoperative 3D CT images reveal satisfactory reduction and fixation of the acetabular fracture. **k** The postoperative X-ray antero-posterior image at 3 months demonstrate that the fracture healing is achieved

Case studies are illustrated in Figs. 5 and 6.

## Discussion

The K-L approach is always recommended in the treatment of acetabular transverse with or without posterior wall fractures [2–5]. Anatomic reduction of the posterior fractures can be achieved intraoperatively under direct vision, and the gap or step at the anterior column and quadrilateral surface are also allowed to palpate through the greater sciatic notch. Therefore, the anterior fractures are possibly reduced by using pelvic reduction tools via the K-L approach. However, every TOAF has a different personality, and in some situations achieving stable fixation of anterior column fracture through a single posterior approach might not be feasible, especially for the T-shaped fractures. No matter which position is chosen, the anterior column fracture gap with the mean of 1.3–2.1 mm usually exists in transverse acetabular fractures after posterior reduction via a single K-L approach [9, 10]. Additionally, simple posterior plate fixation probably leads to opening of the anterior fracture line due to insufficient pre-contouring of the plate. Consequently, it’s critical to reduce and fix the double-column fractures simultaneously. In a series of transverse acetabular fracture models created with the synthetic hemipelvis, the biomechanical study has proved that an anterior column lag screw with posterior plate fixation provided significantly stiffer fixation when residual displacement of anterior column was assessed [14]. Using the finite element analysis method, the results of testing the biomechanical properties of transverse acetabular fractures with different fixation even showed that the posterior plate with an anterior column lag screw was superior to double-column plating [21].

Because the entry point of the antegrade anterior column screw is located at the tilt iliac external crest, the guiding wire is relatively difficult to be inserted accurately. In contrast, the entry point of the retrograde anterior column screw is easier to be positioned, but the working length sometimes is unreasonable due to the position of transverse fracture line. Consequently, the retrograde anterior column screws aren’t suitable for the fixation of transverse acetabular fractures. Some recent studies reported that percutaneous fixation with surgical navigation is increasingly minimally invasive and safe [17, 18, 22]. Robot-aided orthopedic surgery has recently emerged as a more viable tool to enable higher precision surgery, as well as saving surgical time [17, 18]. In certain situations, the available corridor of the anterior column can interfere with the screws that were fixed along with posterior plates, resulting in insufficient space for the cannulated screw. Therefore, accurate surgical planning should be carried out, otherwise the position of posterior plates or screws has to be adjusted.

In this study, compared with the minimally invasive anterior plate fixation, percutaneous anterior column screw fixation has the advantages of short operation time, less invasiveness and fewer complications, although similar imaging and clinical effects can be achieved. Anatomic findings showed that the whole anterior column in male can accommodate a φ6.5-mm cannulated screw very well, while it doesn’t fit all the females unless shorter lag screws were chosen [23]. We did find some female patients with the narrow anterior column, in whom not only the entry points but also the directions had to be planned accurately. All of the cannulated screws were located in the bony corridor of anterior column with robotic assistance, and the mean screw length was 111.3 ± 15.3 cm (range, 85-135 mm). The reason why the results are different with those in the literature is that the guide wire is flexible and the cannulated screw can be inserted like an intramedullary nail. If anterior column screw fixation cannot be successfully performed with free-hand technique, anterior plate fixation is an alternative method for the treatment of TOAFs. Although the anterior column fracture can be exposed clearly via an anterior approach, such as ilioinguinal, stoppa, or para-rectus approach, extensive soft tissue dissection is inevitable. The key to minimally invasive anterior plate fixation used in this study is to establish a subperiosteal tunnel between the medial and lateral windows, and insert a reconstruction locking plate based on limited exposure, to avoid iatrogenic damage, decrease surgical time and reduce intraoperative blood loss. Additionally, another bullet point is to achieve a pre-contoured plate according to the anatomical morphology of the anterior pelvic ring, which can reduce the irritation of the plate to the anterior femoral nerve and vascular bundle. The following procedures are to compress and stabilize the anterior fracture with screw fixation, with the consequence of eliminating the fracture gap. According to the results we summarized, minimally invasive anterior plate fixation is slightly better than screw fixation in correcting the anterior step displacement after posterior fixation. If there are only limited gap without step at the anterior fracture site, percutaneous anterior column screw fixation has obvious advantage on eliminating the fracture gap and can be inserted first.

Since 2000, Mears [24] and Gansslen [25] have reported an excellent reduction rate of 75% (41/55) and 76% (79/104) for the acetabular transverse with posterior wall fractures, respectively. In comparison, the overall excellent reduction rates in this study were 83.3% (10/12) in group A and 82.4% (14/17). The reason why the excellent reduction rates were significantly higher than those reported in literature was that almost all the transverse with or without posterior wall acetabular fractures have achieved anatomic reduction. However, if we recalculate the excellent reduction rate of complicated TOAFs, T-shaped fractures, it will certainly decrease. Of note, the single K-L approach was used completely in group A, which was much higher than Mears [24] with 77% and Gansslen [24] with 90%. The results also indirectly verified the importance of robot navigation that we used in this study.

Admittedly, appropriate indications should be deliberated in treating the complicated TOAFs. If anterior fracture gap were filled with obvious bone callus, it’s challenging for surgeons to deal with via a single K-L approach. In comparison, T-shaped acetabular fractures are more intractable to reduce via the K-L approach due to the separation of both-column, consequently with the lower excellent rate and functional outcomes than the other transverse-oriented fractures. If the residual gap or step of the anterior column is over 1 cm after the reduction and fixation of the posterior portion fractures, it is often inevitable to combine the anterior approach for further management [7].

Additionally, there are still some limitations in this study. The findings may be limited by the small number of patients and a relatively short follow-up time, which may decrease the persuasiveness of the study. Additionally, we did not investigate other important factors that may influence the reduction quality of the acetabular fracture, such as bone mineral density (BMD). Also, robot-aided orthopedic surgery has not been popularized in most medical institutions due to the lack of corresponding equipment.

## Conclusion

It is reasonable to suggest that TOAFs can be managed by posterior fixation via the K-L approach combined with robot-aided anterior column screw fixation if possible. Compared with minimally invasive anterior plate fixation, robot-aided anterior column screw fixation provided the similar reduction and function results, whereas it has significant advantages in surgical time, intraoperative blood loss and invasiveness. Minimally invasive anterior fixation can be an alternative choice for the treatment of TOAFs if the robot system is lacking or a percutaneous anterior column screw is not allowed due to the inappropriate corridor.

## Data Availability

The datasets analyzed during the current study are available from the corresponding author on reasonable request.
